# Ten-Year Piece of Retained Products of Conception: An Unusual Cause of Secondary Infertility

**DOI:** 10.7759/cureus.67337

**Published:** 2024-08-20

**Authors:** Amelie M Harpey, Tori E Abdalla, Bridget McNierney, Emily G Lingo, Ellen G Wood

**Affiliations:** 1 Obstetrics and Gynecology, Philadelphia College of Osteopathic Medicine, Philadelphia, USA; 2 Reproductive Endocrinology and Infertility, IVFMD-South Florida Institute for Reproductive Medicine, Cooper City, USA

**Keywords:** placental remnants, hysteroscopic morcellation, intrauterine mass, infertility, retained products of conception

## Abstract

Retained products of conception (RPOC) occur when the placenta does not properly separate from the uterine wall. The prevalence of RPOC varies between countries and the result of pregnancy, between vaginal delivery, cesarean delivery, and miscarriage or dilation and curettage (D&C). Overall, RPOC has a higher incidence in developed countries where practices tend toward earlier manual removal of the placenta instead of waiting for spontaneous delivery. Typically, retained products following delivery are symptomatic, causing hemorrhage or infection; however, RPOC related to pregnancy termination are most likely asymptomatic. This article describes a case in which a patient presented with 10 years of infertility following the termination of her first pregnancy with D&C. We aim to explore diagnostic modalities, treatment options, and possible complications of long-term RPOC.

## Introduction

Centuries ago, before the innovations of modern-day family planning, Egyptians needed a way to sterilize female camels and prevent pregnancy as they made their trek across the desert. Small stones were placed in the camel’s womb, successfully preventing the camel from carrying offspring until the stones were removed [1]. To this day, this same concept of a foreign body within the uterine cavity preventing pregnancy reigns true, from the development of the copper intrauterine device (IUD) for intentional pregnancy prevention to possible structural causes of infertility, including intrauterine adhesions, fibroids, polyps, and retained products of conception (RPOC) [2]. 

In the third stage of labor, following the delivery of the baby, the placenta is delivered from the uterus. Expected placenta delivery requires adequate uterine contractions, which causes shearing of the placenta from the decidua of the uterus and ends with the expulsion of the tissue [3]. Occasionally, the placenta does not separate from the wall properly, which can result in RPOC. Following vaginal delivery, there are three kinds of RPOC: placenta adherens, trapped placenta, and a partial accreta [4]. Placenta adherences result from a failed muscle wall contraction, called the myometrium, behind the placenta. This type is typically seen in uterine atony [4]. A trapped placenta is detached from the myometrium but cannot exit due to a closed cervix, and a partial accreta occurs when a small portion of the placenta has embedded itself deeper into the myometrium, preventing complete detachment [4]. Risk factors for retained placenta include prolonged oxytocin use, multiparity, preterm delivery, history of uterine surgery, and in vitro fertilization (IVF) conceptions, as well as prior history of retained placenta and congenital uterine anomalies [3]. 

Additionally, placental hypoperfusion disorders, such as pre-eclampsia, have been proposed as mechanisms for retained placental tissue, although a common mechanism has yet to be established [3]. Acute complications of retained placenta can include life-threatening postpartum hemorrhage, endometritis, and the possibility of further retained placenta, which can lead to delayed hemorrhage [3]. Long-term complications include intrauterine adhesions and infertility [5]. 

Retained placenta is diagnosed clinically when the placenta fails to spontaneously separate from the uterine wall following delivery of the baby, although timing seems to vary [3]. However, the diagnosis of additional retained products of conception is best made with transvaginal ultrasound (TVUS) [6]. In the setting of miscarriage or abortion requiring dilation and curettage (D&C), the incidence of RPOC was higher than following a full-term vaginal delivery which has an incidence of 3% [4]. A quality improvement (QI) study showed a significant decrease in the rate of RPOC and the need for reoperation following the implementation of intraoperative imaging using transvaginal ultrasound (TVUS). Prior to the implementation of TVUS, the incidence of RPOC was 6.9%. The incidence within this QI study decreased to 0% with the use of TVUS. Additionally, it was associated with lower rates of reoperation [6].

This case details an asymptomatic woman experiencing infertility after a D&C 10 years prior. In this article, we aim to review the best diagnostic measures, treatments, and complications of RPOC, in addition to other causes of infertility due to uterine etiology.

## Case presentation

**Figure 1:**
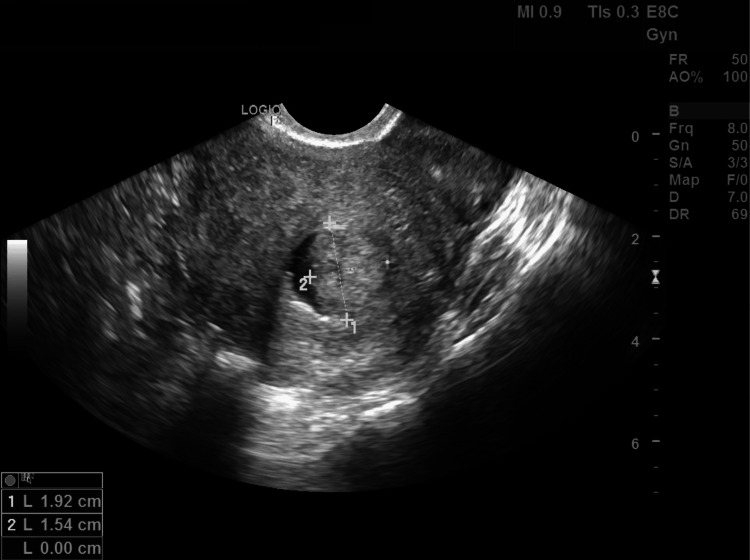
Fundal view of the uterus on saline infusion sonohysterogram, demonstrating a 1.92 cm x 1.54 cm mass in the uterine cavity.

**Figure 2:**
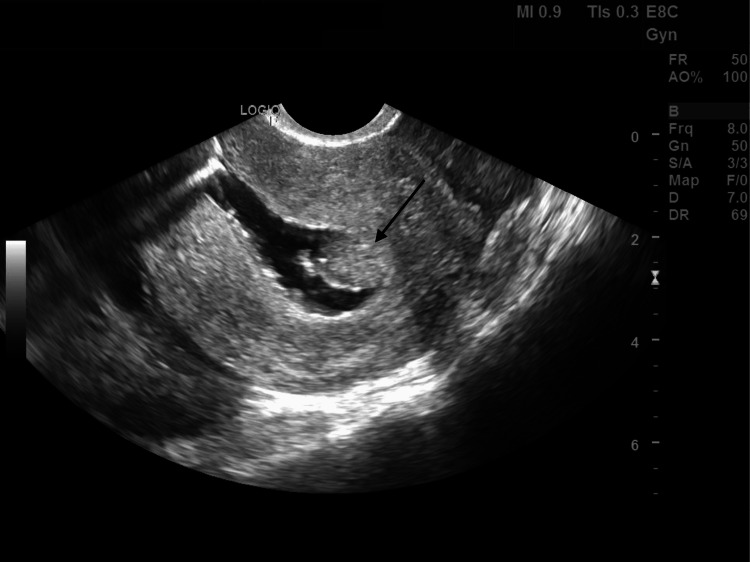
Sagittal view of saline sonohysterogram depicting a mass in the fundus of the uterus (black arrow).

A 36-year-old woman, gravida 1 para 0 (G1P0), presents to the clinic for evaluation of infertility. Her only pregnancy, 10 years prior to presentation, was a termination via D&C at 12 weeks gestation due to anencephaly. Other than this D&C, she denied any previous medical or surgical history, and all other hematologic, serologic, and endocrine investigations were normal. The patient initially presented to another fertility clinic, where she underwent a hysterosalpingogram (HSG) that reported only right-sided tubal patency. During her first visit to our fertility clinic, her transvaginal ultrasound revealed findings suggestive of an intrauterine mass, with the differential diagnosis being a uterine polyp or a submucosal fibroid. The patient subsequently underwent a saline infusion sonohysterogram (SIS), which showed a regular and symmetrical endometrium with a central 2 cm polyp (Figures 1, 2).

**Figure 3:**
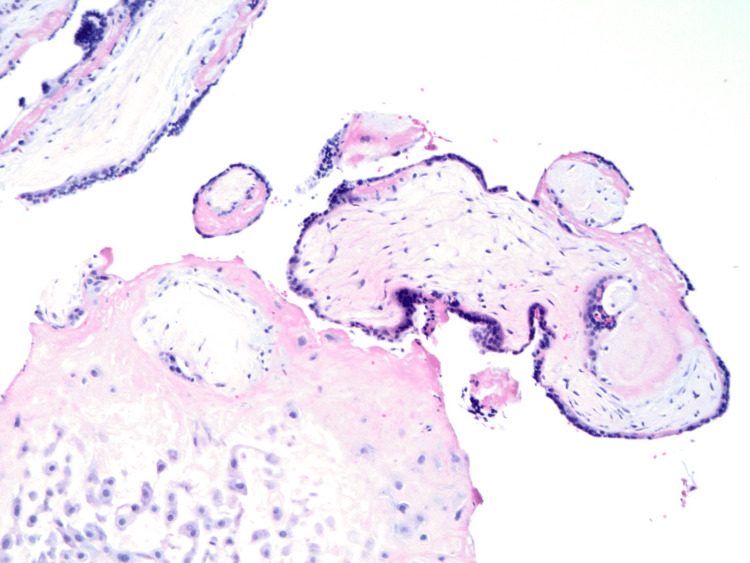
H&E stain at 200x magnification of chorionic villi from the intrauterine mass confirmed the diagnosis of retained products of conception. H&E: hematoxylin and eosin.

**Figure 4:**
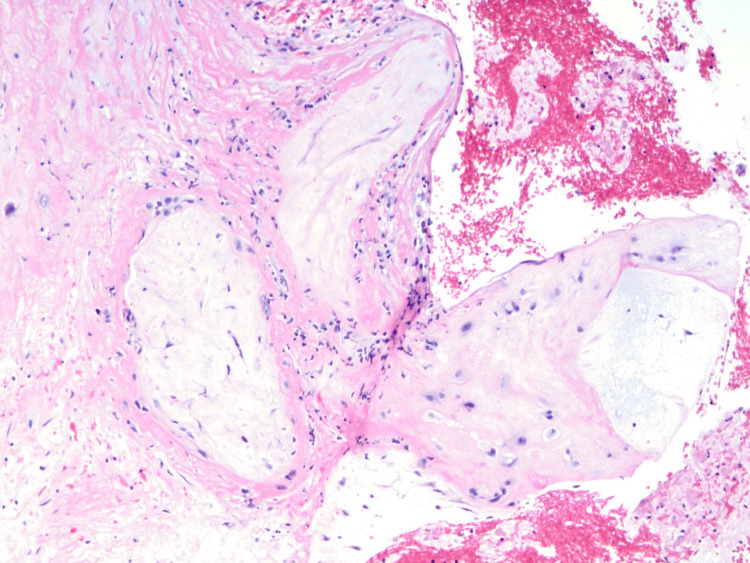
H&E stain at 200x magnification of necrotic, degenerated villi identified within the intrauterine mass. H&E: hematoxylin and eosin.

The patient underwent hysteroscopy with MyoSure removal of the mass previously observed on ultrasound. Intraoperative findings revealed spongy yellow tissue that was resected and removed. Pathological findings described a degenerated and necrotic first-trimester chorionic villi (Figures 3, 4) associated with focally necrotic decidua consistent with retained placental tissue from a recent gestational state.

The patient returned one week after surgery for her postoperative visit with no complaints. A pelvic ultrasound was performed then, and the findings were consistent with a normal uterus. It was recommended that she repeat her HSG after her next menses. The follow-up HSG revealed a normal uterine cavity with normal bilateral tubal patency. Four months after her hysteroscopy, the patient returned to the office, reporting a spontaneous pregnancy.

## Discussion

RPOC is not an uncommon complication of pregnancy. In some, the presence of RPOC may be a differential diagnosis if the patient has a history of abnormal bleeding, pelvic pain, fever, or persistently dilated cervix [5]. Symptoms are more likely to be present in patients who are diagnosed with RPOC after delivery, while asymptomatic RPOC is more common after termination [7]. Following delivery of a full-term pregnancy, symptoms of RPOC often include life-threatening postpartum hemorrhage and endometritis. The presenting symptoms and the structural integrity of the placenta are much more apparent and require immediate treatment. However, in the setting of pregnancy termination, it is difficult to determine whether all the products of conception have been removed from the uterus without further confirmatory testing. In the case of our patient, her primary complaint was infertility, which is nonspecific and, therefore, not immediately correlated with an abortion 10 years prior. One limitation to our timeline is that due to such a length of time after her D&C, it is possible that our patient had an undiagnosed pregnancy and was unaware prior to a subsequent miscarriage; however, this possibility is unlikely due to the size of the mass found within the uterus. 

Occasionally, following pregnancy, a placental site nodule (PSN) can develop and may be confused with RPOC. A PSN is a rare benign lesion of the intermediate trophoblast that represents incomplete involution of the placental implantation site. These lesions typically present with menorrhagia, intermenstrual bleeding, or an abnormal pap smear, but they usually present remotely from the preceding pregnancy. Ultrasound may show an irregular endometrium with a slight increase in vascularity, and it may look like a polyp on hysteroscopy. PSNs can be seen as a filling defect on HSG and SIS, adding to the differential during an infertility workup [8]. The suspected pathogenesis of these lesions is thought to result from endometrial alterations secondary to surgical procedures like C-section and endometrial curettage, leading to abnormal involution of the placental site. Pathologically, they show a microscopic, rounded, well-circumscribed contour with sub-involuted vessels, intermediate trophoblast, and abundant extracellular hyalinized material [8]. 

Several imaging modalities can be used to diagnose RPOC following a failed pregnancy. A QI study on intraoperative imaging showed that transvaginal ultrasound following surgical termination of pregnancy reduced the incidence of RPOC from 6.9% to 0% and reduced reoperations from eight reoperations in 130 patients to zero reoperations in 95 patients [6]. Additionally, a study on using in-office hysteroscopy with ultrasonography in early pregnancy loss (EPL) after IVF showed that office hysteroscopy detected retained products of conception in 24.4% of those with normal pelvic ultrasonography results [9]. The most common symptom that is associated with RPOC following EPL is vaginal bleeding, which is also to be expected following termination of pregnancy. This makes RPOC challenging to discern without additional imaging. A retrospective cohort study on diagnostic accuracy in symptomatic versus asymptomatic patients with RPOC reported that an endometrial thickness greater than 1.49 cm on ultrasound aided the diagnosis and supported an enhanced diagnostic value using Doppler flow in symptomatic women. The study also emphasized the superiority of integrating hysteroscopy in addition to ultrasound, regardless of patients’ symptoms [7]. 

Once RPOC has been identified, removal is indicated, regardless of whether or not the patient is symptomatic. In the past, D&C was the procedure of choice; however, this is associated with a higher risk of intrauterine adhesions, leading to difficulty conceiving in the future [10]. A study on different modalities showed that hysteroscopic removal (HR) and ultrasound-guided electric vacuum aspiration (EVA) of RPOC led to lower risks of intrauterine adhesions and had higher rates of complete removal. The pregnancy rates for HR and EVA were 96.7% and 92.9%, respectively [11]. There are two main kinds of hysteroscopic removal techniques available: electro-resection and morcellation [12]. MyoSure, the type of morcellation technology utilized in this patient’s surgery, uses a dual suction and rotating curettage system that maximizes tissue removal while decreasing operating times compared to electro-resection [13]. The complete resection rate for patients with RPOC is between 94 and 100%, depending on the type of placental adhesion, specifically partial accreta [14]. MyoSure technology may be limited when removing placenta accreta because the device remains parallel to the uterine wall during the procedure, making it challenging to remove lesions that extend into the myometrium [13]. Improvement in pregnancy rate and decreased time to conception were positive outcomes in MyoSure patients. However, live birth rate, abortion rate, and premature birth rate were not found to have a statistically significant difference between MyoSure and electro-resection [13]. 

While pregnancy complications such as preterm delivery, pre-eclampsia, placental abruption, malpresentation, cervical incompetence, first-trimester bleeding, and miscarriage following prior D&C were not found to have increased incidence, there was a significant increase in the rates of postpartum hemorrhage due to endomyometrial injury [15]. An additional study on the use of hysteroscopy for the removal of RPOC confirmed that hysteroscopy was associated with lower intrauterine adhesion rates and incomplete evacuations when compared with dilation and curettage. RPOC associated with early-term and mid-term pregnancy should be surgically removed as early as possible to reduce the risk of bleeding and prevent intrauterine adhesions [16]. A small study including 40 patients with early pregnancy loss evaluated the benefits of adding hysteroscopy to D&C procedures. They found three cases of RPOC immediately after D&C using hysteroscopy that would have been otherwise missed on intraoperative ultrasound [17]. 

While acute complications of RPOC can be life-threatening, the timing of removal has not been shown to have any effect on future reproductive outcomes in a stable patient. Whether the RPOC remains in the uterus for 48 hours or 10 years, there is no significant difference in terms of future pregnancy outcomes once they have been removed [18,19]. While the timing of removal does not affect pregnancy in the future, having RPOC will be a cause of infertility in a woman. RPOC functions similarly to any intrauterine object, whether an IUD, a fibroid, or an endometrial polyp. The patient detailed in this case presentation had unexplained infertility for several years following her initial pregnancy. Once her RPOC had been diagnosed and removed, she was able to conceive spontaneously.

## Conclusions

Retained products of conception can be considered a uterine foreign body and cause infertility if undiagnosed. As seen in the patient presented above, RPOC was found during an infertility workup 10 years following her first pregnancy. Symptomatic RPOC is likely to result in severe complications such as postpartum hemorrhage or endometritis. When asymptomatic, the timing of removal of RPOC does not affect future reproductive outcomes. The patient was able to conceive spontaneously months following her hysteroscopic removal of RPOC with Myosure. The use of TVUS and hysteroscopy following a D&C for pregnancy termination can reduce the incidence of RPOC and the need for reoperation in the future.

## References

[1] Knowles J (2024). Planned parenthood: a history of birth control methods. https://www.plannedparenthood.org/files/2613/9611/6275/History_of_BC_Methods.pdf.

[2] Freytag D, Günther V, Maass N, Alkatout I (2021). Uterine fibroids and infertility. Diagnostics (Basel).

[3] Perlman NC, Carusi DA (2019). Retained placenta after vaginal delivery: risk factors and management. Int J Womens Health.

[4] Weeks AD (2008). The retained placenta. Best Pract Res Clin Obstet Gynaecol.

[5] van den Bosch T, Daemen A, Van Schoubroeck D, Pochet N, De Moor B, Timmerman D (2008). Occurrence and outcome of residual trophoblastic tissue: a prospective study. J Ultrasound Med.

[6] Larish MA, Jensen EC, Mara CK (2021). The implementation of routine procedural transvaginal sonography to decrease retained products of conception: a quality improvement initiative. BMC Womens Health.

[7] Aiob A, Mikhail SM, Sgayer I, Kalendaryov A, Odeh M, Lowenstein L, Sharon A (2024). Diagnostic accuracy and characteristics of symptomatic versus asymptomatic retained products of conception: a retrospective cohort study. Eur J Obstet Gynecol Reprod Biol.

[8] Kim SY, Chang AS, Ratts VS (2005). Radiographic and hysteroscopic findings of a placental site nodule. Fertil Steril.

[9] George JS, Naert MN, Lanes A, Yin S, Bharadwa S, Ginsburg ES, Srouji SS (2023). Utility of office hysteroscopy in diagnosing retained products of conception following early pregnancy loss after in vitro fertilization. Obstet Gynecol.

[10] Hooker AB, Aydin H, Brölmann HA, Huirne JA (2016). Long-term complications and reproductive outcome after the management of retained products of conception: a systematic review. Fertil Steril.

[11] Wagenaar LP, van Vugt WL, Huppelschoten AG (2024). Reproductive and obstetrical outcomes after treatment of retained products of conception: hysteroscopic removal vs ultrasound-guided electric vacuum aspiration, a prospective follow-up study. Am J Obstet Gynecol.

[12] Liang Y, Ren Y, Wan Z, Guo L, Dong J, Chen Y, Lv L (2017). Clinical evaluation of improved MyoSure hysteroscopic tissue removal system for the resection of type II submucosal myomas. Medicine (Baltimore).

[13] Yong J, Wan Y, Ye M (2023). Comparative analysis of the clinical efficacy and reproductive outcomes of the hysteroscopic tissue removal system (MyoSure) and hysteroscopic electroresection in the treatment of benign intrauterine lesions. Int J Gynaecol Obstet.

[14] Georgiou D, Tranoulis A, Jackson TL (2018). Hysteroscopic tissue removal system (MyoSure) for the resection of polyps, sub-mucosal leiomyomas and retained products of conception in an out-patient setting: a single UK institution experience. Eur J Obstet Gynecol Reprod Biol.

[15] Lohmann-Bigelow J, Longo SA, Jiang X, Robichaux AG (2007). Does dilation and curettage affect future pregnancy outcomes?. Ochsner J.

[16] Han L, Shi G, Zheng A, Ruan J (2023). Hysteroscopy for retained products of conception: a single-institution experience. BMC Womens Health.

[17] Moore O, Tzur T, Vaknin Z, Rabbi ML, Smorgick N (2024). Hysteroscopy-assisted suction curettage for early pregnancy loss: does it reduce retained products of conception and postoperative intrauterine adhesions?. Arch Gynecol Obstet.

[18] Tarasov M, Burke YZ, Stockheim D, Orvieto R, Cohen SB (2020). Does the time interval between the diagnosis to hysteroscopic evacuation of retained products of conception affect reproductive outcome?. Arch Gynecol Obstet.

[19] Melcer Y, Smorgick N, Schneider D, Pansky M, Halperin R, Ben-Ami I (2016). Infertility following retained products of conception: does the timing of surgical intervention matter?. Isr Med Assoc J.

